# Supplementary material for: Dynamic transcriptomic profiles of zebrafish gills in response to zinc supplementation
Source: BMC Genomics. 2010 Oct 11;11:553. doi: 10.1186/1471-2164-11-553 (PMC3091702; doi:10.1186/1471-2164-11-553)
Supplement: Additional file 2 — Interactive Direct Interaction Network representing the molecular interactions between zinc, copper, iron, calcium and proteins encoded by transcripts changed by zinc supplementation. Mini web-site containing index.html and hyperlinked pages in subdirectory describing a Direct Interaction Network automatically generated based on curated interactions contained within the proprietary PathwayArchitect database. Ovals represent proteins and the circles symbolize metal ions. Objects are coloured by their abundance in zebrafish at the time-point they were significantly different from the control is a scale from -4 fold (dark green) to +4 fold (dark red). Where significant differences were found at more than one time-point, the colour overlay shows expression at the first instance. Dark blue squares denote 'binding', and light blue squares 'expression'; green squares stand for 'regulation', green diamonds for 'metabolism', and green circles for 'promoter binding'. Arrow heads indicate directionality of the interaction where annotated. All nodes and edges can be further interrogated by selecting the relative area of the image. [file 1471-2164-11-553-S2.zip › PathwayArchitect Zn xs DIN/796330.html]

# BINDING:

|  |  |
| --- | --- |
| Type | BINDING |
| Effect | None |


---

|  |  |
| --- | --- |
| Score | 0 |


---

|  |  |
| --- | --- |
| Reference Count | 108 |


---

|  |  |
| --- | --- |
| Mechanism | Unknown |


---

|  |  |
| --- | --- |
| Reference:0 || Sentence | "Transferrin-bound iron increased P1 promoter activity 2.5-fold and hemin decreased P1 promoter activity, but neither had any effect on P2 activity." |
| PMID | 7610931 |
| Year | 1995 |
| Species | Mouse |
| Journal | J Bone Miner Res |
| RefScore | 0 |
| Source | PArchNLP |
  |
|  |  |


---

|  |  |
| --- | --- |
 Reference:1 || Sentence | "To determine the ability of pyoverdin to compete for iron with the human iron-binding protein transferrin, wild-type P. aeruginosa ATCC 15692 (PAO1 strain) and PAO pyoverdin-deficient mutants were grown at 37 degrees C in bicarbonate-containing succinate medium to which apotransferrin had been added." |
| PMID | 8550201 |
| Year | 1996 |
| Species | Mouse |
|  | Human |
| Journal | Infect Immun |
| RefScore | 2 |
| Source | PArchNLP |
  ||  |  |


---

|  |  |
| --- | --- |
 Reference:2 || Sentence | "Transferrin (TF), a major plasma protein, binds and transports ferric iron." |
| PMID | 2079722 |
| Year | 1990 |
| Species | Human |
|  | Mouse |
| Journal | J Neurosci Res |
| RefScore | 0 |
| Source | PArchNLP |
  ||  |  |


---

|  |  |
| --- | --- |
 Reference:3 || Sentence | "We tested the hypothesis that the toxicity of 6-OHDA is caused by its interaction with serum ferric transferrin (Fe-TF) resulting in release of iron." |
| PMID | 10727233 |
| Year | 2000 |
| Species | Mouse |
|  | Human |
| Journal | Biochemistry |
| RefScore | 1 |
| Source | PArchNLP |
  ||  |  |


---

|  |  |
| --- | --- |
 Reference:4 || Sentence | "The iron-binding plasma protein transferrin (TF) is essential for supplying iron to cells and the prevention of iron toxicity." |
| PMID | 1366420 |
| Year | 1990 |
| Species | Human |
|  | Mouse |
| Journal | SAAS Bull Biochem Biotechnol |
| RefScore | 1 |
| Source | PArchNLP |
  ||  |  |


---

|  |  |
| --- | --- |
 Reference:5 || Sentence | "At low cytosolic iron level, IRE-BP is activated and binds to stem-loop untranslated regions (IRE regions) of transferrin and ferritin mRNAs, activating and inhibiting their translations, respectively." |
| PMID | 7840606 |
| Year | 1995 |
| Species | Mouse |
| Journal | Arch Biochem Biophys |
| RefScore | 0 |
| Source | PArchNLP |
  ||  |  |


---

|  |  |
| --- | --- |
 Reference:6 || Sentence | "The iron-binding protein transferrin has major roles in transporting, delivering, and sequestering ferric ions acquired by body tissues." |
| PMID | 8548503 |
| Year | 1996 |
| Species | Human |
|  | Mouse |
| Journal | J Gerontol A Biol Sci Med Sci |
| RefScore | 1 |
| Source | PArchNLP |
  ||  |  |


---

|  |  |
| --- | --- |
 Reference:7 || Sentence | "Another iron-binding protein, transferrin, stimulated proliferation of HB4C5 cells as well as lactoferrin, but its stimulatory effect on IgM production was negligible." |
| PMID | 1366591 |
| Year | 1990 |
| Species | Human |
|  | Mouse |
| Journal | Cytotechnology |
| RefScore | 1 |
| Source | PArchNLP |
  ||  |  |


---

|  |  |
| --- | --- |
 Reference:8 || Sentence | "Iron added bound to transferrin or as a salt, iron-nitrilotriacetate, can mediate the effect." |
| PMID | 8495498 |
| Year | 1993 |
| Species | Mouse |
| Journal | Cell Immunol |
| RefScore | 0 |
| Source | PArchNLP |
  ||  |  |


---

|  |  |
| --- | --- |
 Reference:9 || Sentence | "The iron-binding growth factor transferrin is taken up and localised in the hindgut of midgestation mouse embryos." |
| PMID | 8875074 |
| Year | 1996 |
| Species | Mouse |
| Journal | Dev Dyn |
| RefScore | 0 |
| Source | PArchNLP |
  ||  |  |


---

|  |  |
| --- | --- |
 Reference:10 || Sentence | "In order to distinguish between a requirement for iron and a possible additional requirement for the iron-binding protein transferrin per se, the ability of mouse lymphocytes to proliferate in response to concanavalin A has been investigated." |
| PMID | 3002966 |
| Year | 1986 |
| Species | Mouse |
|  | Human |
| Journal | Immunology |
| RefScore | 1 |
| Source | PArchNLP |
  ||  |  |


---

|  |  |
| --- | --- |
 Reference:11 || Sentence | "Transferrin (TF) is a major plasma protein that binds ferric iron and transports it to all target tissues of the body." |
| PMID | 2290357 |
| Year | 1990 |
| Species | Mouse |
| Journal | Mech Ageing Dev |
| RefScore | 0 |
| Source | PArchNLP |
  ||  |  |


---

|  |  |
| --- | --- |
 Reference:12 || Sentence | "Immunoprecipitation with a broad spectrum milk antiserum showed that more than 80% of caseins were secreted into the lumina, whereas iron-binding proteins (both lactoferrin and transferrin) were present in comparable amounts in each compartment." |
| PMID | 2806122 |
| Year | 1989 |
| Species | Mouse |
| Journal | Development |
| RefScore | 1 |
| Source | PArchNLP |
  ||  |  |


---

|  |  |
| --- | --- |
 Reference:13 || Sentence | "Transferrins from both homologous and heterologous species were equally effective, but iron-binding half-molecules of transferrin, and low molecular weight iron chelates produced no enhancement." |
| PMID | 7251059 |
| Year | 1981 |
| Species | Mouse |
| Journal | Immunology |
| RefScore | 1 |
| Source | PArchNLP |
  ||  |  |


---

|  |  |
| --- | --- |
 Reference:14 || Sentence | "Addition of iron-binding proteins (human serum transferrin, mouse serum transferrin, human lactoferrin) to the luminal fluid in tied-off segments of mouse intestine in vivo led to reduced 59Fe3+ absorption from 59Fe3+-nitrilotriacetate when compared to 59Fe3+-nitrilotriacetate alone." |
| PMID | 3768410 |
| Year | 1986 |
| Species | Mouse |
| Journal | Biochim Biophys Acta |
| RefScore | 1 |
| Source | PArchNLP |
  ||  |  |


---

|  |  |
| --- | --- |
 Reference:15 || Sentence | "It is likely that aluminium is transported to the brain by the iron-binding protein transferrin and enters the brain via specific transferrin receptors." |
| PMID | 1608913 |
| Year | 1992 |
| Species | Mouse |
| Journal | Pharmacol Toxicol |
| RefScore | 0 |
| Source | PArchNLP |
  ||  |  |


---

|  |  |
| --- | --- |
 Reference:16 || Sentence | "The compound removes iron from complexes with the host iron-binding proteins transferrin and lactoferrin, and the resulting ferric enterochelin is assimilated by the bacterial cell." |
| PMID | 6448022 |
| Year | 1980 |
| Species | Mouse |
| Journal | Antimicrob Agents Chemother |
| RefScore | 0 |
| Source | PArchNLP |
  ||  |  |


---

|  |  |
| --- | --- |
 Reference:17 || Sentence | "The virulent isolates produced high titers of hemolysin, were resistant to inactivation by serum complement, produced phenolate siderophore, and utilized transferrin-bound iron." |
| PMID | 1444386 |
| Year | 1992 |
| Species | Mouse |
|  | Human |
| Journal | Appl Environ Microbiol |
| RefScore | 0 |
| Source | PArchNLP |
  ||  |  |


---

|  |  |
| --- | --- |
 Reference:18 || Sentence | TfR1 (transferrin receptor 1) mediates the uptake of transferrin-bound iron and thereby plays a critical role in cellular iron metabolism. |
| Year | 2005 |
| PMID | 16092918 |
| Species | Human |
| Journal | Biochem J |
| RefScore | 1 |
| Source | PArchNLP |
  ||  |  |


---

|  |  |
| --- | --- |
 Reference:19 || Sentence | In pathological situations, non-transferrin-bound iron, ferritin, and hemoglobin/haptoglobin and heme/hemopexin complexes assume greater importance in iron delivery to the organ. |
| Year | 2005 |
| PMID | 16315136 |
| Species | Human |
| Journal | Semin Liver Dis |
| RefScore | 2 |
| Source | PArchNLP |
  ||  |  |


---

|  |  |
| --- | --- |
 Reference:20 || Sentence | Iron infusion increased total (Delta 601 mug/100 mL, CI 507, 696) and non-transferrin-bound iron (Delta 237.2 mumol/L, CI 173.6, 300.8) approximately 10-fold, as well as redox-active iron nearly five-fold (Delta 0.76 mumol/L, CI 0.54, 0.98). |
| Year | 2005 |
| PMID | 16316358 |
| Species | Human |
| Journal | Kidney Int |
| RefScore | 1 |
| Source | PArchNLP |
  ||  |  |


---

|  |  |
| --- | --- |
 Reference:21 || Sentence | Our data suggest that 300 mg intravenous iron sucrose has a vasodilatory effect, but does not impair vascular reactivity in dialysis patients, despite a significant increase in non-transferrin-bound and redox-active iron. |
| Year | 2005 |
| PMID | 16316358 |
| Species | Human |
| Journal | Kidney Int |
| RefScore | 2 |
| Source | PArchNLP |
  ||  |  |


---

|  |  |
| --- | --- |
 Reference:22 || Sentence | A methodology was developed to study the interaction of DFO and its metabolites with plasma iron pools by stabilizing DFO with aluminum ions, thereby preventing iron shuttling from non-transferrin-bound iron (NTBI) onto DFO after plasma collection. |
| Year | 2005 |
| PMID | 16339661 |
| Species | Human |
| Journal | Ann N Y Acad Sci |
| RefScore | 0 |
| Source | PArchNLP |
  ||  |  |


---

|  |  |
| --- | --- |
 Reference:23 || Sentence | Non-Transferrin-Bound Iron during Blood Transfusion Cycles in {beta}-Thalassemia Major. |
| Year | 2005 |
| PMID | 16339692 |
| Species | Human |
| Journal | Ann N Y Acad Sci |
| RefScore | 0 |
| Source | PArchNLP |
  ||  |  |


---

|  |  |
| --- | --- |
 Reference:24 || Sentence | Serum non-transferrin-bound iron (NTBI) levels assessed at arbitrary time points during transfusion cycles may not be representative if NTBI is undergoing significant changes during transfusion cycles. |
| Year | 2005 |
| PMID | 16339692 |
| Species | Human |
| Journal | Ann N Y Acad Sci |
| RefScore | 0 |
| Source | PArchNLP |
  ||  |  |


---

|  |  |
| --- | --- |
 Reference:25 || Sentence | As an adaptation to the iron-restricted environment of the host, A. pleuropneumoniae possesses iron acquisition pathways mediated by surface receptors that specifically bind transferrin from the host. |
| Year | 2006 |
| PMID | 16005639 |
| Species | Mouse |
| Journal | Protein Expr Purif |
| RefScore | 0 |
| Source | PArchNLP |
  ||  |  |


---

|  |  |
| --- | --- |
 Reference:26 || Sentence | Serum non-transferrin bound iron in hemodialysis patients not receiving intravenous iron. |
| Year | 2005 |
| PMID | 15979061 |
| Species | Human |
| Journal | Clin Chim Acta |
| RefScore | 0 |
| Source | PArchNLP |
  ||  |  |


---

|  |  |
| --- | --- |
 Reference:27 || Sentence | BACKGROUND: Non-transferrin bound iron (NTBI) has been found to be raised in end stage renal disease (ESRD) patients on hemodialysis (HD) receiving intravenous (IV) iron. |
| Year | 2005 |
| PMID | 15979061 |
| Species | Human |
| Journal | Clin Chim Acta |
| RefScore | 0 |
| Source | PArchNLP |
  ||  |  |


---

|  |  |
| --- | --- |
 Reference:28 || Sentence | Bicarbonate inhibits the growth of Staphylococcus epidermidis in platelet concentrates by lowering the level of non-transferrin-bound iron. |
| Year | 2005 |
| PMID | 16271102 |
| Species | Human |
| Journal | Transfusion |
| RefScore | 1 |
| Source | PArchNLP |
  ||  |  |


---

|  |  |
| --- | --- |
 Reference:29 || Sentence | BACKGROUND: Platelet concentrates (PCs) contain non-transferrin-bound iron (NTBI) owing to the displacement of iron from plasma-derived transferrin by citrate. |
| Year | 2005 |
| PMID | 16271102 |
| Species | Human |
| Journal | Transfusion |
| RefScore | 0 |
| Source | PArchNLP |
  ||  |  |


---

|  |  |
| --- | --- |
 Reference:30 || Sentence | The transferrins (TF) are a family of bilobal glycoproteins that tightly bind ferric iron. |
| Year | 2005 |
| PMID | 16300393 |
| Species | Human |
| Journal | Biochemistry |
| RefScore | 1 |
| Source | PArchNLP |
  ||  |  |


---

|  |  |
| --- | --- |
 Reference:31 || Sentence | Nonetheless, intravenous iron therapy has effects on endothelial cells, polymorphonuclear leucocytes and cytokines which are most likely related to non-transferrin bound labile iron. |
| Year | 2005 |
| PMID | 16281958 |
| Species | Human |
| Journal | Eur J Clin Invest |
| RefScore | 1 |
| Source | PArchNLP |
  ||  |  |


---

|  |  |
| --- | --- |
 Reference:32 || Sentence | Steap3 is expressed highly in hematopoietic tissues, colocalizes with the Tf cycle endosome and facilitates Tf-bound iron uptake. |
| Year | 2005 |
| PMID | 16227996 |
| Species | Mouse |
| Journal | Nat Genet |
| RefScore | 1 |
| Source | PArchNLP |
  ||  |  |


---

|  |  |
| --- | --- |
 Reference:33 || Sentence | These results confirm the previous finding that Fe-Tf and HFE compete for the receptor by binding to an overlapping site on the TfR helical domain. |
| Year | 2003 |
| PMID | 14691533 |
| Species | Human |
| Journal | PLoS Biol |
| RefScore | 1 |
| Source | PArchNLP |
  ||  |  |


---

|  |  |
| --- | --- |
 Reference:34 || Sentence | Non-transferrin-bound serum iron (NTBI) in megaloblastic anemia: effect of vitamin B(12) treatment. |
| Year | 2004 |
| PMID | 14745427 |
| Species | Human |
| Journal | Hematol J |
| RefScore | 0 |
| Source | PArchNLP |
  ||  |  |


---

|  |  |
| --- | --- |
 Reference:35 || Sentence | Brain capillary endothelium and choroid plexus epithelium regulate transport of transferrin-bound and free iron into the rat brain. |
| Year | 2004 |
| PMID | 14756801 |
| Species | Rat |
| Journal | J Neurochem |
| RefScore | 2 |
| Source | PArchNLP |
  ||  |  |


---

|  |  |
| --- | --- |
 Reference:36 || Sentence | We conclude that transport of circulating Tf-bound and free iron could be equally important for its delivery to the CNS. |
| Year | 2004 |
| PMID | 14756801 |
| Species | Rat |
| Journal | J Neurochem |
| RefScore | 2 |
| Source | PArchNLP |
  ||  |  |


---

|  |  |
| --- | --- |
 Reference:37 || Sentence | Moreover, data suggest that entry of Tf-bound and free iron into the CNS is determined by (i) its initial sequestration by brain capillaries and choroid plexus, and (ii) subsequent controlled and slow release from vascular structures into brain interstitial fluid and CSF. |
| Year | 2004 |
| PMID | 14756801 |
| Species | Rat |
| Journal | J Neurochem |
| RefScore | 1 |
| Source | PArchNLP |
  ||  |  |


---

|  |  |
| --- | --- |
 Reference:38 || Sentence | We added dilutions of ferric gluconate, iron sucrose and each of two iron dextran preparations to serum in vitro, passed the resulting samples through alumina columns to remove iron agent and free organic iron, and measured Tf-bound iron in the resulting eluates. |
| Year | 2004 |
| PMID | 14767009 |
| Species | Human |
| Journal | Nephrol Dial Transplant |
| RefScore | 1 |
| Source | PArchNLP |
  ||  |  |


---

|  |  |
| --- | --- |
 Reference:39 || Sentence | Comparing results to serum samples without added iron, we calculated delta Tf-bound iron for each agent at each concentration. |
| Year | 2004 |
| PMID | 14767009 |
| Species | Human |
| Journal | Nephrol Dial Transplant |
| RefScore | 1 |
| Source | PArchNLP |
  ||  |  |


---

|  |  |
| --- | --- |
 Reference:40 || Sentence | Finally, we compared delta Tf-bound iron to the concentration of added agent and calculated the percent iron donation to Tf. |
| Year | 2004 |
| PMID | 14767009 |
| Species | Human |
| Journal | Nephrol Dial Transplant |
| RefScore | 1 |
| Source | PArchNLP |
  ||  |  |


---

|  |  |
| --- | --- |
 Reference:41 || Sentence | RESULTS: We found that Tf-bound iron increased with added iron concentration for each agent: delta Tf-bound iron was directly related to the concentration and type of iron agent (P<0.001). |
| Year | 2004 |
| PMID | 14767009 |
| Species | Human |
| Journal | Nephrol Dial Transplant |
| RefScore | 0 |
| Source | PArchNLP |
  ||  |  |


---

|  |  |
| --- | --- |
 Reference:42 || Sentence | Oxygen free radicals are produced only by free iron, but stored body iron markers reflect iron bound to ferritin or transferrin, which are produced to sequester catalytically active free iron. |
| Year | 2004 |
| PMID | 14975519 |
| Species | Human |
| Journal | Med Hypotheses |
| RefScore | 1 |
| Source | PArchNLP |
  ||  |  |


---

|  |  |
| --- | --- |
 Reference:43 || Sentence | However, some recent studies find non-transferrin bound iron (NTBI) or the intracellular labile iron pool (LIP) in the presence of triggers disturbing iron homeostasis, such as alcohol consumption. |
| Year | 2004 |
| PMID | 14975519 |
| Species | Human |
| Journal | Med Hypotheses |
| RefScore | 0 |
| Source | PArchNLP |
  ||  |  |


---

|  |  |
| --- | --- |
 Reference:44 || Sentence | Role of transient receptor potential canonical 6 (TRPC6) in non-transferrin-bound iron uptake in neuronal phenotype PC12 cells. |
| Year | 2004 |
| PMID | 14640978 |
| Species | Human |
|  | Rat |
| Journal | Biochem J |
| RefScore | 1 |
| Source | PArchNLP |
  ||  |  |


---

|  |  |
| --- | --- |
 Reference:45 || Sentence | Cells take up transferrin-bound iron or NTBI (non-transferrin-bound iron). |
| Year | 2004 |
| PMID | 14640978 |
| Species | Human |
|  | Rat |
| Journal | Biochem J |
| RefScore | 1 |
| Source | PArchNLP |
  ||  |  |


---

|  |  |
| --- | --- |
 Reference:46 || Sentence | We investigated the effect of free, non-transferrin-bound iron occurring in haematological stem cell transplant patients on growth of Staphylococcus epidermidis in serum in vitro, and prevention of bacterial growth by exogenous apotransferrin. |
| Year | 2004 |
| PMID | 15040393 |
| Species | Human |
| Journal | FEMS Immunol Med Microbiol |
| RefScore | 1 |
| Source | PArchNLP |
  ||  |  |


---

|  |  |
| --- | --- |
 Reference:47 || Sentence | Identification of small molecule inhibitors that distinguish between non-transferrin bound iron uptake and transferrin-mediated iron transport. |
| Year | 2004 |
| PMID | 15123270 |
| Species | Human |
| Journal | Chem Biol |
| RefScore | 2 |
| Source | PArchNLP |
  ||  |  |


---

|  |  |
| --- | --- |
 Reference:48 || Sentence | Using this approach, we screened the National Cancer Institute's Diversity Set library for inhibitors of non-transferrin bound iron uptake. |
| Year | 2004 |
| PMID | 15123270 |
| Species | Human |
| Journal | Chem Biol |
| RefScore | 1 |
| Source | PArchNLP |
  ||  |  |


---

|  |  |
| --- | --- |
 Reference:49 || Sentence | Like DMT1, SFT function has been implicated in transferrin and nontransferrin-bound iron uptake. |
| Year | 2004 |
| PMID | 15139022 |
| Species | Mouse |
|  | Human |
| Journal | J Neurosci Res |
| RefScore | 2 |
| Source | PArchNLP |
  ||  |  |


---

|  |  |
| --- | --- |
 Reference:50 || Sentence | Non-transferrin-bound iron in the serum of hemodialysis patients who receive ferric saccharate: no correlation to peroxide generation. |
| Year | 2004 |
| PMID | 15153577 |
| Species | Human |
| Journal | J Am Soc Nephrol |
| RefScore | 0 |
| Source | PArchNLP |
  ||  |  |


---

|  |  |
| --- | --- |
 Reference:51 || Sentence | Assays were performed for both total iron and transferrin-bound iron, from which drug-bound iron could be calculated. |
| Year | 2004 |
| PMID | 15162891 |
| Species | Human |
| Journal | Pharmacotherapy |
| RefScore | 1 |
| Source | PArchNLP |
  ||  |  |


---

|  |  |
| --- | --- |
 Reference:52 || Sentence | CONCLUSIONS: Iron derived from SFGC appears to be rapidly transferred to a bioavailable iron compartment as transferrin-bound iron after digestion in the RES. |
| Year | 2004 |
| PMID | 15162891 |
| Species | Human |
| Journal | Pharmacotherapy |
| RefScore | 0 |
| Source | PArchNLP |
  ||  |  |


---

|  |  |
| --- | --- |
 Reference:53 || Sentence | HFE affects the interaction of transferrin bound iron with transferrin receptors (TfR) thereby modulating iron uptake. |
| Year | 2004 |
| PMID | 15173932 |
| Species | Mouse |
| Journal | J Mol Med |
| RefScore | 1 |
| Source | PArchNLP |
  ||  |  |


---

|  |  |
| --- | --- |
 Reference:54 || Sentence | The first involves binding of iron to transferrin and uptake via transferrin receptors. |
| Year | 2004 |
| PMID | 15039637 |
| Species | Human |
| Journal | Med Sci Monit |
| RefScore | 1 |
| Source | PArchNLP |
  ||  |  |


---

|  |  |
| --- | --- |
 Reference:55 || Sentence | RESULTS: Proinflammatory cytokines increase transferrin and non-transferrin bound iron uptake into human monocytes and increase transferrin-bound iron uptake by synovial fibroblasts, but have no effect on non-transferrin bound uptake into fibroblasts. |
| Year | 2004 |
| PMID | 15039637 |
| Species | Human |
| Journal | Med Sci Monit |
| RefScore | 1 |
| Source | PArchNLP |
  ||  |  |


---

|  |  |
| --- | --- |
 Reference:56 || Sentence | The aims of this study were to investigate the degradation of human transferrin by gingipain cysteine proteinases of P. gingivalis and to demonstrate the production of toxic hydroxyl radicals (HO\*) catalyzed by the iron-containing transferrin fragments generated or by release of iron itself. |
| Year | 2004 |
| PMID | 15271890 |
| Species | Human |
| Journal | Infect Immun |
| RefScore | 0 |
| Source | PArchNLP |
  ||  |  |


---

|  |  |
| --- | --- |
 Reference:57 || Sentence | CONCLUSION: Ethanol exposure induced the transferrin receptor expression, partially through the activation of iron regulatory protein, and increased the transferrin-bound iron uptake in rat hepatocyte cultures. |
| Year | 2004 |
| PMID | 15318094 |
| Species | Rat |
| Journal | Alcohol Clin Exp Res |
| RefScore | 0 |
| Source | PArchNLP |
  ||  |  |


---

|  |  |
| --- | --- |
 Reference:58 || Sentence | BACKGROUND: Transferrin binds extracellular iron and protects tissues from iron-induced oxidative stress. |
| PMID | 15292463 |
| Year | 2004 |
| Species | Human |
| Journal | Nephrol Dial Transplant |
| RefScore | 0 |
| Source | PArchNLP |
  ||  |  |


---

|  |  |
| --- | --- |
 Reference:59 || Sentence | No differences were detected in blood indexes of iron status (including serum ferritin, transferrin saturation, and non-transferrin-bound iron) or in blood lipids or transaminases, but heterozygotes had significantly greater, although normal, fasting glucose concentrations than did wild-type control subjects. |
| PMID | 15447900 |
| Year | 2004 |
| Species | Human |
| Journal | Am J Clin Nutr |
| RefScore | 0 |
| Source | PArchNLP |
  ||  |  |


---

|  |  |
| --- | --- |
 Reference:60 || Sentence | Melanotransferrin (MTf) or melanoma tumor antigen p97 is a membrane-bound transferrin (Tf) homologue that binds iron (Fe). |
| PMID | 15469901 |
| Year | 2004 |
| Species | Mouse |
|  | Human |
|  | Rat |
| Journal | Biochim Biophys Acta |
| RefScore | 1 |
| Source | PArchNLP |
  ||  |  |


---

|  |  |
| --- | --- |
 Reference:61 || Sentence | Iron may populate distinct hepatocellular iron pools that differentially regulate expression of proteins such as ferritin and transferrin receptor (TfR) through iron-regulatory mRNA-binding proteins (IRPs), and may additionally regulate uptake and accumulation of non-transferrin-bound iron (NTBI). |
| PMID | 15543932 |
| Year | 2004 |
| Species | Human |
| Journal | Mol Cell Biochem |
| RefScore | 1 |
| Source | PArchNLP |
  ||  |  |


---

|  |  |
| --- | --- |
 Reference:62 || Sentence | In this study we studied 12 preterm infants in whom the plasma levels of Btot, total hydroperoxide (TH), protein SH groups, HO activity, non-transferrin-bound iron (NTBI), and erythrocyte CuZn superoxide dismutase (CuZn SOD) activity were concurrently measured when the Btot was >220 microM and after a Btot drop of >34 microM. |
| PMID | 15470195 |
| Year | 2004 |
| Species | Human |
| Journal | Pediatr Res |
| RefScore | 0 |
| Source | PArchNLP |
  ||  |  |


---

|  |  |
| --- | --- |
 Reference:63 || Sentence | METHODS AND RESULTS: Addition of 10 mumol/L non-transferrin-bound iron to the incubation medium caused a 2-fold increase in monocyte adhesion to human umbilical vein endothelial cells (HUVECs). |
| PMID | 15486315 |
| Year | 2004 |
| Species | Human |
| Journal | Arterioscler Thromb Vasc Biol |
| RefScore | 0 |
| Source | PArchNLP |
  ||  |  |


---

|  |  |
| --- | --- |
 Reference:64 || Sentence | CONCLUSIONS: Non-transferrin-bound iron increases the level of intracellular labile iron, which promotes monocyte recruitment to endothelium and may thereby contribute to the pathogenesis of atherosclerosis. |
| PMID | 15486315 |
| Year | 2004 |
| Species | Human |
| Journal | Arterioscler Thromb Vasc Biol |
| RefScore | 0 |
| Source | PArchNLP |
  ||  |  |


---

|  |  |
| --- | --- |
 Reference:65 || Sentence | Transferrin receptor 2 (TfR2) is a type 2 transmembrane protein expressed in hepatocytes that binds iron-bound transferrin (Tf). |
| PMID | 15319290 |
| Year | 2004 |
| Species | Human |
| Journal | Blood |
| RefScore | 1 |
| Source | PArchNLP |
  ||  |  |


---

|  |  |
| --- | --- |
 Reference:66 || Sentence | However, TfR2 does not increase when non-Tf-bound iron (FeNTA) or apo Tf is added to the medium. |
| PMID | 15319290 |
| Year | 2004 |
| Species | Human |
| Journal | Blood |
| RefScore | 2 |
| Source | PArchNLP |
  ||  |  |


---

|  |  |
| --- | --- |
 Reference:67 || Sentence | Mouse HFE inhibits Tf-uptake and iron accumulation but induces non-transferrin bound iron (NTBI)-uptake in transformed mouse fibroblasts. |
| PMID | 15389541 |
| Year | 2005 |
| Species | Mouse |
|  | Human |
| Journal | J Cell Physiol |
| RefScore | 1 |
| Source | PArchNLP |
  ||  |  |


---

|  |  |
| --- | --- |
 Reference:68 || Sentence | The activation of a non-transferrin bound iron-uptake pathway that functions in the stable mHFE-transfected clones could explain their normal growth curves and survival. |
| PMID | 15389541 |
| Year | 2005 |
| Species | Mouse |
|  | Human |
| Journal | J Cell Physiol |
| RefScore | 1 |
| Source | PArchNLP |
  ||  |  |


---

|  |  |
| --- | --- |
 Reference:69 || Sentence | The proteins and mechanisms responsible for additional iron uptake into specific regions, or perhaps the redistribution are unclear though the data are supportive of a non-transferrin-bound iron uptake pathway. |
| PMID | 15578731 |
| Year | 2005 |
| Species | Mouse |
| Journal | J Neurosci Res |
| RefScore | 0 |
| Source | PArchNLP |
  ||  |  |


---

|  |  |
| --- | --- |
 Reference:70 || Sentence | This paper describes a comparison of two analytical techniques, one employing bathophenanthrolinedisulfonate (BPT), a most commonly-used reagent for Fe (II) determination, as chromogen and an electrothermal atomic absorption spectroscopy (ETAAS) for the quantification of non-transferrin bound iron (NTBI) in sera from thalassemic patients. |
| PMID | 15916113 |
| Year | 2004 |
| Species | Human |
| Journal | Southeast Asian J Trop Med Public Health |
| RefScore | 1 |
| Source | PArchNLP |
  ||  |  |


---

|  |  |
| --- | --- |
 Reference:71 || Sentence | Neisseria meningitidis, a causative agent of bacterial meningitis and septicemia, obtains transferrin-bound iron by expressing two outer membrane-located transferrin-binding proteins, TbpA and TbpB. |
| PMID | 15664936 |
| Year | 2005 |
| Species | Human |
| Journal | Infect Immun |
| RefScore | 1 |
| Source | PArchNLP |
  ||  |  |


---

|  |  |
| --- | --- |
 Reference:72 || Sentence | Labile plasma iron (LPI) represents a component of non-transferrin-bound iron (NTBI) that is both redox-active and chelatable, capable of permeating into organs and inducing tissue iron overload. |
| PMID | 15737890 |
| Year | 2005 |
| Species | Human |
| Journal | Best Pract Res Clin Haematol |
| RefScore | 1 |
| Source | PArchNLP |
  ||  |  |


---

|  |  |
| --- | --- |
 Reference:73 || Sentence | Earlier studies found that in HeLa cells, HFE expression lowers Tf-mediated iron uptake; here we show that HFE lowers non-Tf-bound iron in TRVb cells and add to a growing body of evidence that HFE may play different roles in different cell types. |
| PMID | 15528318 |
| Year | 2005 |
| Species | Human |
| Journal | Blood |
| RefScore | 1 |
| Source | PArchNLP |
  ||  |  |


---

|  |  |
| --- | --- |
 Reference:74 || Sentence | Effects of intravenous polymaltose iron on oxidant stress and non-transferrin-bound iron in hemodialysis patients. |
| PMID | 15640610 |
| Year | 2005 |
| Species | Human |
| Journal | Nephron Clin Pract |
| RefScore | 1 |
| Source | PArchNLP |
  ||  |  |


---

|  |  |
| --- | --- |
 Reference:75 || Sentence | Serum iron status, non-transferrin-bound iron (NTBI) and markers of oxidative stress were studied in blood samples from these patients. |
| PMID | 15640610 |
| Year | 2005 |
| Species | Human |
| Journal | Nephron Clin Pract |
| RefScore | 1 |
| Source | PArchNLP |
  ||  |  |


---

|  |  |
| --- | --- |
 Reference:76 || Sentence | Serum transferrin (Tf) contains two iron (Fe)-binding sites and plays a vital role in Fe transport. |
| PMID | 15716025 |
| Year | 2005 |
| Species | Mouse |
|  | Human |
| Journal | Biochim Biophys Acta |
| RefScore | 1 |
| Source | PArchNLP |
  ||  |  |


---

|  |  |
| --- | --- |
 Reference:77 || Sentence | Labile plasma iron (LPI) represents the redox active component of non-transferrin-bound iron (NTBI). |
| PMID | 15671444 |
| Year | 2005 |
| Species | Human |
| Journal | Blood |
| RefScore | 1 |
| Source | PArchNLP |
  ||  |  |


---

|  |  |
| --- | --- |
 Reference:78 || Sentence | Results of an international round robin for the quantification of serum non-transferrin-bound iron: Need for defining standardization and a clinically relevant isoform. |
| PMID | 15907869 |
| Year | 2005 |
| Species | Human |
| Journal | Anal Biochem |
| RefScore | 0 |
| Source | PArchNLP |
  ||  |  |


---

|  |  |
| --- | --- |
 Reference:79 || Sentence | Non-transferrin-bound iron (NTBI) appears in the circulation of patients with iron overload. |
| PMID | 15907869 |
| Year | 2005 |
| Species | Human |
| Journal | Anal Biochem |
| RefScore | 2 |
| Source | PArchNLP |
  ||  |  |


---

|  |  |
| --- | --- |
 Reference:80 || Sentence | In turn, Al did induce upregulation of non-Tf bound Fe (NTBI) uptake. |
| PMID | 16085060 |
| Year | 2005 |
| Species | Human |
| Journal | Biochim Biophys Acta |
| RefScore | 1 |
| Source | PArchNLP |
  ||  |  |


---

|  |  |
| --- | --- |
 Reference:81 || Sentence | Divalent metal transporter 1 (DMT1) and transferrin receptor (TfR) might play a key role in non-transferrin-bound iron (NTBI) and transferrin-bound iron (Tf-Fe) uptake by neuronal cells. |
| PMID | 16137791 |
| Year | 2005 |
| Species | Rat |
| Journal | Neurochem Int |
| RefScore | 1 |
| Source | PArchNLP |
  ||  |  |


---

|  |  |
| --- | --- |
 Reference:82 || Sentence | However, this apparent inappropriate regulation of hepcidin correlated with increased transferrin saturation and levels of diferric transferrin in the plasma, which in turn resulted from the reduced capacity of hbd animals to effectively use transferrin-bound iron. |
| PMID | 16239432 |
| Year | 2006 |
| Species | Mouse |
| Journal | Blood |
| RefScore | 1 |
| Source | PArchNLP |
  ||  |  |


---

|  |  |
| --- | --- |
 Reference:83 || Sentence | Intravenous iron significantly increased transferrin saturation and non-transferrin-bound iron (NTBI) when compared with placebo. |
| PMID | 16395269 |
| Year | 2006 |
| Species | Human |
| Journal | Kidney Int |
| RefScore | 1 |
| Source | PArchNLP |
  ||  |  |


---

|  |  |
| --- | --- |
 Reference:84 || Sentence | Iron concentration in the liver and non-transferrin-bound iron were measured in patients only. |
| PMID | 16545687 |
| Year | 2006 |
| Species | Human |
| Journal | Free Radic Biol Med |
| RefScore | 1 |
| Source | PArchNLP |
  ||  |  |


---

|  |  |
| --- | --- |
 Reference:85 || Sentence | In humans, iron-deficiency results in anemia, while excess iron can lead to organ damage as a result of a build-up of non-transferrin-bound iron (NTBI). |
| PMID | 16540419 |
| Year | 2006 |
| Species | Human |
| Journal | Hemoglobin |
| RefScore | 1 |
| Source | PArchNLP |
  ||  |  |


---

|  |  |
| --- | --- |
 Reference:86 || Sentence | A 10-year follow-up shows a marked decrease in the concentration of serum ferritin, non-transferrin-bound iron (NTBI), liver iron and normal hemoglobin (Hb), which allows the patient to reach and maintain a good quality of life. |
| PMID | 16540426 |
| Year | 2006 |
| Species | Human |
| Journal | Hemoglobin |
| RefScore | 0 |
| Source | PArchNLP |
  ||  |  |


---

|  |  |
| --- | --- |
 Reference:87 || Sentence | Melanotransferrin (MTf) or tumor antigen p97 is a transferrin homolog that binds one iron (Fe) atom and has been suggested to play roles in a variety of processes, including Fe metabolism, eosinophil differentiation, and plasminogen activation. |
| PMID | 16291590 |
| Year | 2006 |
| Species | Mouse |
| Journal | Blood |
| RefScore | 1 |
| Source | PArchNLP |
  ||  |  |


---

|  |  |
| --- | --- |
 Reference:88 || Sentence | Accordingly, we investigated the effect of crp mutation on the expression of the vulnibactin-mediated iron-uptake system and the ability of V. vulnificus to utilize transferrin-bound iron, and thus to grow in cirrhotic ascites, a human ex vivo system. |
| PMID | 16553865 |
| Year | 2006 |
| Species | Human |
| Journal | FEMS Microbiol Lett |
| RefScore | 0 |
| Source | PArchNLP |
  ||  |  |


---

|  |  |
| --- | --- |
 Reference:89 || Sentence | Moreover, the crp mutant could not utilize transferrin-bound iron, and its growth was severely suppressed both on transferrin-bound iron and in cirrhotic ascites. |
| PMID | 16553865 |
| Year | 2006 |
| Species | Human |
| Journal | FEMS Microbiol Lett |
| RefScore | 1 |
| Source | PArchNLP |
  ||  |  |


---

|  |  |
| --- | --- |
 Reference:90 || Sentence | These results indicate that crp mutation attenuates the ability to grow on transferrin-bound iron and in a human body fluid by down-regulating the vulnibactin-mediated iron-uptake system. |
| PMID | 16553865 |
| Year | 2006 |
| Species | Human |
| Journal | FEMS Microbiol Lett |
| RefScore | 0 |
| Source | PArchNLP |
  ||  |  |


---

|  |  |
| --- | --- |
 Reference:91 || Sentence | Non-transferrin-bound iron and risk of coronary heart disease in postmenopausal women. |
| PMID | 16618820 |
| Year | 2006 |
| Species | Human |
| Journal | Circulation |
| RefScore | 1 |
| Source | PArchNLP |
  ||  |  |


---

|  |  |
| --- | --- |
 Reference:92 || Sentence | Common presence of non-transferrin-bound iron among patients with type 2 diabetes. |
| PMID | 16644642 |
| Year | 2006 |
| Species | Human |
| Journal | Diabetes Care |
| RefScore | 0 |
| Source | PArchNLP |
  ||  |  |


---

|  |  |
| --- | --- |
 Reference:93 || Sentence | Because vitamin C may cause oxidative stress in the presence of redox active iron, we hypothesized that non-transferrin-bound iron (NTBI), a form of iron susceptible to redox activity, may be present in patients with type 2 diabetes. |
| PMID | 16644642 |
| Year | 2006 |
| Species | Human |
| Journal | Diabetes Care |
| RefScore | 1 |
| Source | PArchNLP |
  ||  |  |


---

|  |  |
| --- | --- |
 Reference:94 || Sentence | Glycemic control, serum iron, proteins involved in iron homeostasis, plasma iron-binding antioxidant capacity in a liposomal model, and non-transferrin-bound iron were measured in 40 type 1 and 67 type 2 diabetic patients compared to 100 nondiabetic healthy control subjects. |
| PMID | 16678014 |
| Year | 2006 |
| Species | Human |
| Journal | Free Radic Biol Med |
| RefScore | 1 |
| Source | PArchNLP |
  ||  |  |


---

|  |  |
| --- | --- |
 Reference:95 || Sentence | Good correlation of non-transferrin-bound iron (NTBI) and TBARs (r=0.8, p<0.01) in LDL strongly supported the contention that iron overload is responsible for initiating the lipid peroxidation in beta-thal/Hb E. |
| PMID | 16681427 |
| Year | 2006 |
| Species | Human |
| Journal | Clin Chem Lab Med |
| RefScore | 1 |
| Source | PArchNLP |
  ||  |  |


---

|  |  |
| --- | --- |
 Reference:96 || Sentence | Uptake of iron is critical for E. histolytica growth and iron-bound human transferrin (holo-transferrin) has been shown to serve as an iron source in vitro. |
| PMID | 16458294 |
| Year | 2006 |
| Species | Human |
| Journal | Exp Parasitol |
| RefScore | 1 |
| Source | PArchNLP |
  ||  |  |


---

|  |  |
| --- | --- |
 Reference:97 || Sentence | Serum non-transferrin-bound iron and low-density lipoprotein oxidation in heterozygous hemochromatosis. |
| PMID | 16682004 |
| Year | 2006 |
| Species | Human |
| Journal | Biochem Biophys Res Commun |
| RefScore | 1 |
| Source | PArchNLP |
  ||  |  |


---

|  |  |
| --- | --- |
 Reference:98 || Sentence | Liver hepcidin mRNA levels correlated with hemoglobin concentration and inversely correlated with serum transferrin receptor, erythropoietin and non-transferrin-bound iron. |
| PMID | 16769583 |
| Year | 2006 |
| Species | Human |
| Journal | Haematologica |
| RefScore | 1 |
| Source | PArchNLP |
  ||  |  |


---

|  |  |
| --- | --- |
 Reference:99 || Sentence | Iron mobilization from transferrin and non-transferrin-bound-iron (NTBI) can be used to optimize existing chelation therapy protocols for the treatment of iron loaded patients. |
| PMID | 16798643 |
| Year | 2006 |
| Species | Human |
| Journal | Hemoglobin |
| RefScore | 1 |
| Source | PArchNLP |
  ||  |  |


---

|  |  |
| --- | --- |
 Reference:100 || Sentence | Non-transferrin-bound iron (NTBI) detected in thalassemic plasma is highly toxic and chelatable. |
| PMID | 16798656 |
| Year | 2006 |
| Species | Human |
| Journal | Hemoglobin |
| RefScore | 1 |
| Source | PArchNLP |
  ||  |  |


---

|  |  |
| --- | --- |
 Reference:101 || Sentence | When alveolar macrophages were loaded with transferrin-bound (55)Fe, the subsequent release of (55)Fe was inhibited significantly by LPS. |
| PMID | 16648237 |
| Year | 2006 |
| Species | Mouse |
| Journal | Am J Physiol Lung Cell Mol Physiol |
| RefScore | 1 |
| Source | PArchNLP |
  ||  |  |


---

|  |  |
| --- | --- |
 Reference:102 || Sentence | Serum transferrin reversibly binds iron in each of two lobes and delivers it to cells by a receptor-mediated, pH-dependent process. |
| PMID | 16793765 |
| Year | 2006 |
| Species | Human |
| Journal | J Biol Chem |
| RefScore | 1 |
| Source | PArchNLP |
  ||  |  |


---

|  |  |
| --- | --- |
 Reference:103 || Sentence | Immunoprecipitation with transferrin antibodies on brains from Belgrade rats revealed lower uptake of transferrin-bound (59)Fe. |
| PMID | 16879716 |
| Year | 2006 |
| Species | Human |
|  | Rat |
| Journal | J Neurochem |
| RefScore | 1 |
| Source | PArchNLP |
  ||  |  |


---

|  |  |
| --- | --- |
 Reference:104 || Sentence | Zip14 (Slc39a14) mediates non-transferrin-bound iron uptake into cells. |
| PMID | 16950869 |
| Year | 2006 |
| Species | Mouse |
|  | Human |
| Journal | Proc Natl Acad Sci U S A |
| RefScore | 1 |
| Source | PArchNLP |
  ||  |  |


---

|  |  |
| --- | --- |
 Reference:105 || Sentence | We analyzed the capability of Zip14 to mediate non-transferrin-bound iron (NTBI) uptake by overexpressing mouse Zip14 in HEK 293H cells and Sf9 insect cells. |
| PMID | 16950869 |
| Year | 2006 |
| Species | Mouse |
|  | Human |
| Journal | Proc Natl Acad Sci U S A |
| RefScore | 1 |
| Source | PArchNLP |
  ||  |  |


---

|  |  |
| --- | --- |
 Reference:106 || Sentence | A significant 2-fold elevation of non-transferrin bound iron (NTBI) was observed in thalassaemia relative to SCD. |
| PMID | 17010049 |
| Year | 2006 |
| Species | Human |
| Journal | Br J Haematol |
| RefScore | 1 |
| Source | PArchNLP |
  ||  |  |


---

|  |  |
| --- | --- |
 Reference:107 || Sentence | Myeloablative conditioning prior to allogeneic stem cell transplantation causes a rapid increase in transferrin saturation and potentially toxic non-transferrin-bound iron (NTBI) in plasma. |
| PMID | 16925790 |
| Year | 2006 |
| Species | Human |
| Journal | Br J Haematol |
| RefScore | 1 |
| Source | PArchNLP |
  |


---

|  |  |
| --- | --- |
